# Stem Cells as *In Vitro* Model of Parkinson's Disease

**DOI:** 10.1155/2012/980941

**Published:** 2012-04-30

**Authors:** Patricia L. Martínez-Morales, Isabel Liste

**Affiliations:** Unidad de Regeneración Neural, Área de Biología Celular y del Desarrollo, Centro Nacional de Microbiología, Instituto de Salud Carlos III (ISCIII), Majadahonda, 28220 Madrid, Spain

## Abstract

Progress in understanding neurodegenerative cell biology in Parkinson's disease (PD) has been hampered by a lack of predictive and relevant cellular models. In addition, the lack of an adequate *in vitro* human neuron cell-based model has been an obstacle for the uncover of new drugs for treating PD. The ability to generate induced pluripotent stem cells (iPSCs) from PD patients and a refined capacity to differentiate these iPSCs into DA neurons, the relevant disease cell type, promises a new paradigm in drug development that positions human disease pathophysiology at the core of preclinical drug discovery. Disease models derived from iPSC that manifest cellular disease phenotypes have been established for several monogenic diseases, but iPSC can likewise be used for phenotype-based drug screens in complex diseases for which the underlying genetic mechanism is unknown. Here, we highlight recent advances as well as limitations in the use of iPSC technology for modelling PD “in a dish” and for testing compounds against human disease phenotypes *in vitro*. We discuss how iPSCs are being exploited to illuminate disease pathophysiology, identify novel drug targets, and enhance the probability of clinical success of new drugs.

## 1. Introduction

Parkinson's disease (PD) is the second most common neurodegenerative disorder, characterized by a large number of motor and nonmotor features that can affect function in a variable degree.

The main pathological hallmark in PD is the loss of midbrain dopaminergic (DA) neurons in the substantia nigra pars compacta (SNpc) projecting to the striatum and abnormal cytoplasmic inclusions enriched in *α*-synuclein, the Lewy bodies, deposited in surviving neurons of the brain [1–3].

There is no effective test for the diagnosis of PD; the disorder must be diagnosed based on clinical criteria. The main clinical features are tremor at rest (unilateral, prominent in the distal part of an extremity), rigidity (increased resistance to move), akinesia or bradykinesia (slowness of movement), postural instability, and other motor abnormalities. Other symptoms include secondary motor symptoms such as dystonia and dysphagia and nonmotor symptoms including cognitive abnormalities, sleep disorders, and pain [3].

Despite the research efforts in this area, with new and intriguing findings constantly being reported, at present, PD is still an incurable disease, but treatment can improve quality of life and functional capacity. To date, L-dopa in combination with a peripheral dopa decarboxylase inhibitor (benserazide or carbidopa) is the most effective therapy as an initial treatment option. However, not all symptoms respond equally to the drug; while tremor may be only marginally reduced, bradykinesia and rigidity respond better. Unfortunately, the treatment's success is reduced over time, and side effects increase, leaving the patient helpless [2]. Deep brain stimulation of the subthalamic nuclei is an additional therapeutic option for PD patients but requires surgical intervention.

Although all of these treatments provide symptomatic relief, none of them is able to stop or reverse the progression of the disease [1, 4]; for this reason there is a need for novel therapeutic approaches. One alternative strategy is cell-replacement therapy; in fact, clinical trials with intrastriatal transplantation of human embryonic mesencephalic tissue have shown that grafted DA neurons reinnervate the striatum, restore the striatal dopamine release, and, in some patients, induce a major clinical benefit [5–7].

## 2. Molecular and Cellular Mechanism of Parkinson's Disease

The cause of PD is still unclear but most people suffering this disorder have idiopathic PD (around 90%). A small proportion of cases (approximately 10%), however, can be attributed to known genetic factors that contribute to PD complex pathogenesis.

Our understanding of the mechanisms underlying the initiation and progression of PD began with the identification of mutations in the gene encoding *α*-synuclein (SNCA) and the demonstration that *α*-synuclein is the major component of Lewy bodies, present in the disease. Since then, at least 16 loci (designed as *PARK1* to *PARK16*) and 11 genes have been associated with inherited forms of parkinsonism, including, for example, *PARK1, PARK4/SNCA*, *PARK2/parkin, PARK5/ubiquitin COOH-terminal hydrolase L1* (*UCHL1*), *PARK6/PTEN-induced kinase 1 (PINK1), PARK/DJ-1, *and* PARK8/Leucine-rich repeat kinase 2 (LRRK2)*.

*SNCA* is an autosomal dominant gene that encodes the protein *α*-synuclein, expressed abundantly in presynaptic terminals of the neurons [8]. Several evidences support the physiological functions of *α*-synuclein in the regulation of vesicle dynamics at the presynaptic membrane [9]. Mutations in *SNCA* increase in the self-assembly and fibrillization of the protein that might lead to the formation of the pathogenic inclusion bodies [9]. Another autosomal dominant gene implicated in PD disease is the leucine-rich repeat kinase 2 (*LRRK2*) [8, 10]. *LRRK2* encodes a large protein with multiple domains, including a Ras-like GTP binding domain and a serine, threonine kinase domain [10]. Mutations within these two functional domains have been associated with PD [8, 10]. In normal conditions, the function of LRRK2 kinase had been implicated in the regulation of the cytoskeleton architecture [10]. In contrast, *Parkin* is an autosomal recessive gene involved in PD [11]. This gene encodes the Parkin protein with an ubiquitin-like sequence E3, which acts as a substrate for target proteins bound to degrade by the ubiquitin proteasome system (UPS) [11]. Inactivation of Parkin leads to reduction in UPS-mediated degradation of target proteins [11] that could result in protein accumulation. In addition, some data suggest a possible function of Parkin in mitochondria, where the protein is localized and promotes gene transcription [8, 11]. *PINK1* is another autosomal recessive gene, whose mutations might cause PD [11]. *PINK1* encodes a protein localized in the mitochondria membrane and its function is associated with protection of cells from stress-induced mitochondrial dysfunction [8, 11]. Interestingly, mutants of *Drosophila melanogaster* lacking *PINK1* display phenotypes similar with those *Parkin* mutants; moreover the forced expression of Parkin1 is able to rescue the mitochondrial dysfunction caused by the absence of *PINK1*, suggesting their interaction [9, 11]. Likewise, DJ-1 is a protein localized in the mitochondria membrane and mutations in this gene may cause autosomal recessive early-onset PD [8, 11]. Its functions are related to the resistance of oxidative stress [11].

The knowledge acquired of these proteins has revealed pathways of neurodegeneration that may be shared between inherited and sporadic PD. A set of data in different model systems strongly suggest that mitochondrial dysfunction plays a central role in clinically similar, early-onset autosomal recessive PD forms caused by *parkin *and *PINK1* and possibly *DJ-1 *gene mutations [12, 13]. Further comprehension of molecular and cellular mechanisms and interaction between these proteins that causes PD with others is essential to identify crucial and potential targets to improve the treatment.

## 3. The Importance of *In Vitro* Models of PD

Most of the current knowledge about neurological diseases, including PD, is gathered from postmortem studies due to the limitations of live brain tissue. This restricts the understanding of the disease progression and development, since postmortem samples only represent the end-stage of the disease. In addition, aspects of the exhibited pathology in these samples could be secondary and not faithfully reflect the exact disease phenotype on a cellular level. Besides, interspecies differences make it difficult to accurately simulate human neurological diseases in animal models. Therefore, disease modelling by recapitulating the diseases phenotype *in vitro* and in defined cell populations is an important advancement and would make it possible to understand cellular and molecular mechanisms of the neurodegenerative disorder [14, 15]. Consider that investigation of a multifactorial disease, such as PD, is more challenging than monogenic disorders due to their complex genetic backgrounds and because they are usually influenced by environmental factors [15].

A progressive loss of substantia nigra DA neurons is the main pathological hallmark of PD. Understanding the mechanism of neuronal cell death involved in PD may be of value in developing neuroprotective therapies. However studying neuronal cell death in human brains is extremely difficult by several (methodological, practical) reasons. Development of *in vitro* models of DA neurons can be powerful, as they would allow the study of neurodegeneration as well as novel therapeutic strategies [16]. Nevertheless, availability of human DA neurons derived from fetal material is extremely limited, and it has been difficult to examine directly toxicity and/or protective effects of multiple factors in these neurons.

**Figure 1:**
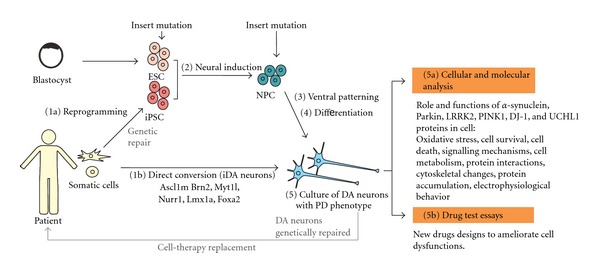
Possible cellular sources for modeling Parkinson's disease *in vitro*. Somatic cells from patient with PD can be reprogrammed into iPSC and differentiated into mesencephalic dopaminergic neurons (1a)–(5). ESC and NPC can be genetically modified by inserting specific mutations related with PD and be differentiated. Alternatively, somatic cells can be directly converted into dopaminergic neurons (1b).

In this context, stem cells, particularly pluripotent stem cells and neural stem/progenitor cells, are an excellent source of cells, because of their availability, unlimited proliferation, and plasticity to differentiate into other cell types. Moreover, stem cells are an excellent alternative to *ex vivo* primary cultures or established immortalized cell lines that can contribute to our understanding of neuronal neurodegenerative process and our ability to analyze the cytotoxic or neuroprotective effects of chemicals, drugs, and so forth (Figure 1).

## 4. Stem Cell Types and Properties

Stem cells are characterized by the ability to renew themselves through mitotic cell division and differentiate into a diverse range of specialized cell types. They can be classified according to their potential to differentiate into specialized cells. The first type is totipotent stem cells that can give rise to an entire viable organism, including placental cells. The zygote and the cells at the very early stages following fertilization (i.e., the 2-cell stage) are considered totipotent.

The second type is pluripotent stem cells, which have the capacity to develop into specialized cells of the three germ layers (ectoderm, mesoderm, and endoderm) except extraembryonic tissues, such as placenta. The first and best described are Embryonic Stem Cells (ESCs), derived from the inner cell mass of the blastocyst [17]. Theoretically, because of their properties, these cells may constitute an optimal source of DA neurons for cell-replacement therapies and drug screening experiments; however, to achieve this aim, it is essential to have an efficient protocol for differentiation into functional midbrain DA neurons. In fact, cultures enriched in human DA neurons have been generated from ESC using a variety of methods, such as the use of the coculture with stromal cells, growth factors, secreted factors, transcription factors, and morphogens, with some beneficial effects having been demonstrated after transplantation of these cells in animal models of PD [18–21].

Recent advances in stem cell biology have led to technologies to reprogram somatic cells from the adult human to a state of pluripotency [22–24]. The first reported lines of reprogrammed cells, termed induced pluripotent stem cells (iPSCs), were originally generated by introducing four transcription factors: *Oct3/4*, *Sox2*, *Klf4,* and *c-myc* (or *Nanog*, *Lin28*) into adult fibroblasts. These cells are similar to hESC in morphology, gene expression profile, and differentiation potential. The induced iPSC technology offers new possibilities for biomedical research and clinical applications, as these cells could be used as an *in vitro* cellular model of PD, and for autologous transplantation (theoretically, no immunosuppressive therapy would be necessary). For this reason, it is essential to obtain an efficient and strict differentiation protocol of hiPSC into midbrain DA-like neurons. In addition, hiPSC do not raise ethical concerns since they are derived from somatic cells, following routine tissue donation procedures.

The third type of stem cells is multipotent stem cells that only generate specific lineages of cells, like Neural Stem Cells (NSCs) that are derived from neural tissues. These cells are self-renewing and differentiate into lineage-specific neural precursor or progenitor cells (NPCs) that can give rise to all cell types (neurons, astrocytes, and oligodendrocytes) of the nervous system [25]. However, although sometimes not evident from the literature, hNSCs—particularly those derived from the ventral mesencephalon (vm)—grow poorly in culture, their properties change over time (passages), and they lose their ability to generate neurons, particularly DA neurons, thus making them difficult to use on a large-scale approach [25, 26].

## 5. Directed Differentiation of Pluripotent Stem Cells into DA Neurons

The necessary first step towards PD modeling is the production, in enough number, of disease neuronal phenotypes, that is, DA neurons, from differentiated human pluripotent stem cells *in vitro*. Current *in vitro* differentiation from either ESC or iPSC includes protocols based on embryoid body formation or the use of stromal feeder coculture [18–20, 27–34]. Efficient generation of DA neurons needs the combined actions of factors such as Noggin, FGF8, Sonic Hedgehog, Retinoic Acid, Wnt1, BNDF, GNDF, Ascorbic Acid, cyclic-AMP, and Wnt5 [18, 20, 27, 29], similar to those secreted factors present during development [35]. *In vitro*, early exposure to Noggin [20, 27], antagonist of the BMP signaling, or to inhibitors of Lefty/Activin/TGF*β* pathways allows a highly efficient feeder-free neural induction in adherent cultures and permits a dopaminergic and motoneuronal potential [18, 20]. In contrast, neural induction can be obtained in the absence of factors and coculturing ES with stromal feeder cell lines [28]. Subsequently, dopaminergic patterning is established by the combined action of FGF8, FGF2, SHH [18, 20], GNDF [28], BDNF, and ascorbic acid. Finally, terminal differentiation is accomplished after withdrawal of SHH and FGF8 and promoted by the presence of ascorbic acid, GDNF, TGFb-1, cyclic-AMP, and Wnt5 [29].

Alternatively, DA neurons can be obtained by the forced expression of transcription factors crucial for ventral midbrain identity [36–40]. Thus overexpression of *Lmx1a *induces DA neurons from murine ESC [36, 37], hESC, and hiPSC [41]; moreover, neuron precursors derived from hESC overexpressing *Lmx1a* are able to survive and differentiate when grafted into the brain of adult mice [41].

Although iPSC and ESC differ in their origin, differentiation of both cell types into DA neurons seems to use similar cues and signals. The analysis by transcriptome revealed no differences in the level of expression of genes involved in dopaminergic differentiation such as *EN1*, *Nurr1*, *TH*, *AADC*, and *Girk2*; moreover analysis of genes involved in imprinting, cell cycle regulation, and reprogramming revealed no significant differences [34].

## 6. Induced Pluripotent Stem Cells as *In Vitro* Model of PD

Derivation of pluripotent stem cells from somatic tissues has provided researchers with a source of patient-specific stem cells. In addition, iPSC technology renders a good model *in vitro* for diseases and drug treatment essays [42].

So far, some groups have developed protocols to increase the yield of DA neurons generation from iPSC from either human or mice [22, 30–32, 42, 43]. In murine models, following protocols developed for ES cells, neural precursor cells and DA neurons were obtained from healthy iPSC [33]. Moreover, derived cells transplanted into the developing brain are able to integrate, migrate, differentiate, and display electrophysiological functions showing spontaneous action potential currents in the host brain. Cells derivatives included glutamatergic, GABAergic, and DA neurons. Importantly, grafts derived from iPSC are capable to restore motor function in animal models for PD [33] suggesting that dopaminergic neurons derived from iPSC are functional *in vivo*.

In humans, DA neurons derived from iPSC can be obtained from healthy donors [34] or patients with PD caused by idiopathic conditions [32, 42] or by mutation [30, 31]. For instance, DA neurons can be generated from iPSCs that carry a mutation in *LRRK2* gene (p.G2019S), the most common PD-related mutation [8, 10]. After reprogramming, culture differentiation protocol using feeders, iPSC-p.G2019S generated a significant number of DA neurons up to 55 days of differentiation; furthermore, these neurons show properties of mature neurons, including the expression of synaptotagmin-1, a protein localized to synaptic vesicles, and the ability of fire action potentials in response to depolarizing current injections and produce spontaneous synaptic activities. Moreover these DA neurons are able to synthesize and release dopamine in response to stimulation with high potassium [30]. A detailed phenotypic characterization related with the PD at day 35 reveals that iPSC-p.G2019S expressed higher levels of genes involved in oxidative stress pathways than controls; indeed trials testing the peroxide-induced cell death show that G2019S-iPSC-derived DA neurons may be more susceptible to oxidative stress and show significantly more cell death than controls. Due to the phenotypes in iPSC-p.G2019S resemblance to the PD phenotype that provides a good model for the *in vitro* disease, this system has been used to test some potential drugs for the treatment of PD [30].

In contrast, other models of PD based on triplication of *α*-synuclein locus had been generated [31]. This mutation causes a fully penetrant and aggressive form of PD with dementia [8–10] compared with the homozygous G2019S mutation of *LRRK2* that has incomplete penetrance, even with homozygous conditions [44]. Using a feeder-free monolayer differentiation method, iPSC differentiated efficiently into midbrain DA neurons after 20 to 31 days when *α*-synuclein protein could be detected and secreted to media [31]. In addition to this model, fibroblasts obtained from a patient carrying the A53T (G209A) *α*-synuclein mutation have been reprogrammed into iPSC and successfully differentiated into DA neurons [43], which could serve as a good model for the *in vitro* analysis; nevertheless, further phenotypic characterization of cells related with PD remains to be studied. 

Other models include those DA neurons derived from iPSC and obtained from patient with idiopathic conditions [32, 42]. After reprogramming patient iPSC, cells were differentiated into DA neurons using the stromal feeder cell-based differentiation protocol. At 42 days, these cells, including DA and non-DA neurons, were transplanted into the striatum of healthy animals. 8 weeks after implantation DA neurons marked with the nigral marker Girk-2 were found into the viable grafts. Moreover, transplantation experiments by engrafting DA neurons derived from iPSC on animal models of PD showed functional effects, although only a few of them sent their axons toward the DA-depleted host striatum. Analysis of behavior in animal models of PD exhibited a significant improvement of motor dysfunction [32, 34]. In summary, several evidences suggest that DA neurons from pluripotent stem cells are functional in both *in vitro* and *in vivo* conditions. Hence, some of the DA neurons derived from PD patients that exhibit some characteristic phenotypes of the disease could provide a valuable cellular source to study *in vitro* the PD. For instance, iPSCs derived from PD patients carrying a nonsense (c.1366C>T; p.*Q*456X) or missense (c.509T>G; p.V170G) mutations in the *PINK1* gene have been used to examine the role of endogenous PINK1 in dopaminergic neurons [45]. *PINK1* encodes a kinase localized on the outer mitochondrial membrane and is implicated in the regulation of mitochondrial degradation [11]; mutations in *PINK1* have been associated with PD [8, 11]. In contrast, Parkin proteins function as an E3 ubiquitin ligase and are localized in the cytosol [11]. In addition, Parkin can be translocated to damaged mitochondria in a PINK1-dependent manner [44]. Thus, experiments on DA neurons from PD patients exhibit impairment in the translocation of Parkin of mutant *PINK1* iPSC cell-derived DA neurons compared to controls. Moreover, rescue experiments by overexpressing wild type PINK1 in *PINK1* mutant neurons restore the translocation of Parkin to mitochondria [45]. In conclusion, DA neurons obtained *in vitro* from PD patients are a suitable model to study the pathogenesis of PD at cellular level (Figure 1; Table 1).

However, several challenges must be overcome before successful implementation of iPSC-based drug screening and pathway discovery can be achieved (Figure 1). The most critical issue is whether the PD phenotype can be reproduced *in vitro*, and if so, whether it can accurately predict disease behavior *in vivo*. PD can be difficult to model, since it occurs late in life and is caused by complex environmental and genetic factors. In fact, in one study that generated DA neurons from iPSC derived from patients with sporadic PD, no obvious abnormalities could be detected [42], indicating that additional stressors may be required to reveal the disease phenotype. Nevertheless, the study of rare family forms of the disease that are associated with specific gene mutations can provide valuable information on the general disease mechanisms. It would be interesting to study whether the iPSC generated from familial PD patients could exhibit disease genotypes and phenotypes *in vitro.*

Other additional limitations are related to the low efficiency and high variability of the reprogramming process and the heterogeneity of the maturation stage and cellular phenotypes obtained after differentiation of iPSC into DA neurons. Even though great progress has been made, our understanding of the factors controlling the induction and specification of DA neuronal fate is far for complete. Further advances in the field will facilitate the generation of clinically relevant DA neurons at least *in vitro*.

## 7. Direct Conversion of Somatic Cells to DA Neurons

Recent reports have demonstrated that human somatic cells can be directly converted to functional neurons, named induced neurons (iNs) by using combined expression of defined factors (*Ascl1, Brn2, and Myt1l*) [39]; the same authors showed that these neurons can be directed toward dopaminergic phenotype by overexpression of *Lmx1a* and *FoxaA2* (two genes involved in DA neuron generation during development). A different cocktail of factors, with only three transcription factors (*Mash1, Nurr1,* and *Lmx1a*), were used by other group for direct generation of functional DA neurons (iDA, induced dopamine neurons) from adult fibroblasts from healthy donors and PD patients [38]. Reprogrammed cells were similar to brain DA neurons in gene expression and dopamine release. However the possible PD phenotype of the generated iDA from PD patients remains to be demonstrated.

This strategy opens new possibilities for regenerative therapies and diseases modelling of PD. Cells generated via direct conversion do not pass through a pluripotent or progenitor state, are probably not tumorigenic, and may serve as an interesting alternative to iPSCs for generating patient and/or disease-specific neurons. However, to be clinically relevant, the overall cell conversion process needs to be highly efficient in order to obtain enough amounts of cells available to study the disease or grafting studies. Both iPSC and iDA cells circumvent the ethical concerns related to embryonic stem cell derivation and potential issues of allogenic rejection in cell-replacement therapy studies.

## 8. Future Prospects

Many questions that define the underlying genesis of the neuronal death in disorders like PD remain unanswered, with evidence suggesting a key role for mitochondrial dysfunction. In this sense, stem cells, in general, and mainly pluripotent stem cells can provide an unlimited source of human DA neurons for *in vitro* studies of neurotoxic and neuroprotective processes that might be related to PD.

iPSC technology has been shown to be of specific interest in monogenic diseases, providing innovative models to understand disease pathology. Modelling late-onset and multifactorial diseases, such as PD, may be more difficult and probably will require additional advances. However, the study or rare forms of PD, associated with specific gene mutations, can provide valuable information on the general disease mechanism. Importantly, patient cell donors can be genetically modified in order to correct mutations. This modification permits the generation of healthy and mutated DA neurons from the same donor, improving the comparative analysis between both cell types in isogenic conditions. Indeed, genetic repairment in iPSC could provide also a good tool in the advances toward iPSC-based cell-replacement therapies.

Even though the large body of current research iPSC technology is still in its infancy, several limitations need to be solved in the near future, for example, standardization in order to obtain medically relevant cells, avoiding contact with animal products, and improvement of reprogramming methods in order to increase efficiency and homogeneity and to avoid tumorigenic properties of iPSC.

Some of these limitations could be circumvent with another innovative approach, “direct reprogramming” of somatic cells from patients to specific neurons (iN). Rapid and efficient generation of patient-specific DA neurons through direct reprogramming may yield many advantages in the screening of pharmaceutical compounds as well as cellular material for analysis of molecular pathways of the disease and for transplantation studies.

##  Conflict of Interests

The authors declare that they have no conflict of interests.

## Figures and Tables

**Table 1:** Examples of *in vitro *models to study Parkinson's disease derived from patient iPSC.

Gene	Genetic disease	Genetic alteration	References
*SNCA *	Autosomal dominant	Triplication of *α*-synuclein locus	[31]
		G209A mutation	[43]
*LRRK2*	Autosomal dominant	p.G2019S mutation	[30]
*PINK1*	Autosomal recessive	p.Q456X and p.V170G mutation	[45]
Idiopathic condition	Idiopathic		[32, 42]
